# Evaluation of compressed sodium chloride on the inactivation of SARS-CoV-2 and surrogates

**DOI:** 10.1371/journal.pone.0277881

**Published:** 2022-11-21

**Authors:** Sudha Bhavanam, Bonita Lee, Yuanyuan Qiu, Nathan Zelyas, Xiaoli Lilly Pang

**Affiliations:** 1 Department of Laboratory Medicine and Pathology, University of Alberta, Edmonton, Alberta, Canada; 2 Department of Pediatrics, University of Alberta, Edmonton, Canada; 3 Public Health Laboratory, Alberta Precision Laboratories, Edmonton, Alberta, Canada; Children’s National Hospital, George Washington University, UNITED STATES

## Abstract

Severe acute respiratory syndrome coronavirus-2 (SARS-CoV-2) causes the global COVID-19 pandemic. Limited studies have been performed on various types of disinfectants utilized to control the spread of this highly contagious virus. This study aimed to investigate the inactivation of SARS-CoV-2 using compressed sodium chloride (CSC) surface. A real-time reverse transcriptase quantitative PCR (RT-qPCR) assay was used to evaluate the effectiveness of CSC on the disintegration of viral RNA in a time dependent manner. The effects of CSC on viral infectivity were determined using a TCID50 assay of a surrogate virus, hCoV-229E, in MRC-5 cell culture. The results demonstrated that CSC achieved a 2 to 3- log_10_ reduction of viral genomic RNA for a laboratory strain of hCoV-229E, and clinical samples of hCoV-229E and hCoV-OC43. A 3 to 4-log_10_ reduction was observed for SARS-CoV-2 (RdRp and E gene) suggesting that a CSC surface could effectively disintegrate the SARS-CoV-2 RNA genome. CSC was observed to have a 6 log_10_ inactivation of infectious hCoV-229E using cell culture after 5 minutes of exposure compared to the control, indicating good disinfection efficacy of a CSC surface against virus.

## Introduction

Severe acute respiratory syndrome coronavirus-2 (SARS-CoV-2) [1] is a novel coronavirus that was first reported in Wuhan, China, and has caused an unprecedented pandemic of coronavirus disease 2019 (COVID-19) [2]. SARS-CoV-2 is an enveloped single stranded RNA virus which belongs to the betacoronavirus genus [3]. The new and emerging SARS-CoV-2 is the seventh recognized coronavirus causing illness in human besides coronavirus 229E, OC43, NL63, HKU1, the more pathogenic severe acute respiratory syndrome coronavirus (SARS-CoV) discovered in 2002, and the Middle East respiratory syndrome coronavirus (MERS-CoV) in 2012 [4]. The genomic profile of SARS-CoV-2 is different from that of SARS-CoV and MERS-CoV [4]. It has been reported that the survival time of SARS-CoV-2 on different surfaces varies from a few minutes to 1 month [5], depending on a number of factors such as the amount of virus inoculated, temperature, surface types, pressure, and relative humidity. SARS-CoV-2 is highly infective, and is easily transmissible from person to person with a high reproduction number [5]. Several approaches have been used to contain SARS-CoV-2, but none of the approaches have been effective when used in isolation. Although vaccines are now available [6,7], there are still significant challenges to achieving global population-wide immunization within a short period of time. Moreover, emerging variants of SARS-CoV-2 have become an issue of increasing concern in the control and management of COVID-19 pandemic. Many countries endured multiple outbreaks of COVID-19 attributed mainly to the emergence of COVID-19 variants of concern (https://www.who.int/en/activities/tracking-SARS-CoV-2-variants).

While there are recommended treatments for severe COVID-19 using different therapeutic agent under study, morbidity and mortality caused by COVID-19 and its impact on healthcare system and society at large is severe [8,9]. The World Health Organization (WHO) has recommended the use of disinfectants as a good approach in preventing and controlling the spread of infection [10,11]. Therefore, one of critical preventive measures against COVID-19 is to inactivate SARS-CoV-2 effectively on environmental surfaces. Previously, the surface made of compressed sodium chloride (CSC) was shown to possess antimicrobial effects against bacteria and fungi upon contact [12,13].

To our knowledge, this is the first study to examine antiviral effects of CSC surface. Using sensitive molecular diagnostic assays, we aim to measure the stability of viral RNA of SARS-CoV-2, laboratory strain of human coronavirus 229E (hCoV-229E) as well as hCoV-229E and hCoV-OC34 detected in clinical samples after varying time of exposure to CSC in comparison with sterile polystyrene (plastic petri dishes routinely used for viral culture) and other types of surfaces including stainless steel and copper. The time-dependent effect of CSC on viral infectivity was examined using viral culture and TCID50 of a laboratory hCoV-229E strain. Since hCoV-229E has 79% genomic similarity with SARS-CoV-2 [14], it was used as the surrogate virus in this study to understand the inactivation by CSC.

## Materials and methods

### Ethics statement

This study was approved by the University of Alberta Health Research Ethics Committee (Pro00118067) and all methods were performed in accordance with institutional guidelines and regulations. Participant consent was waived by the ethics committee as only anonymized and to-be-discarded residual clinical samples were used in this study.

### Testing materials

Four solid material surfaces were used in this study, including CSC, sterile polystyrene (plastic petri dishes routinely used for viral culture), copper, and stainless steel. Sterile polystyrene was used as a baseline control testing surfaces. NSF food safety grade SAE 304 stainless steel (purchased from metal supermarket, Edmonton) and sheets of copper alloy (C70600) containing 84.7% copper gifted from Olin Brass (Louisville Kentucky, USA), which are registered as antimicrobial with the United States Environmental Protection Agency (reg. # 85353–5), were used to compare with CSC. CSC contains 97.5–100% sodium chloride (table salts) in the form of blocks (50mm x50mm x8mm) and possess smooth, non-porous surface which is manufactured via a special compression procedure using salts as a solo raw material (Cargill). The CSC blocks can be molded to create counter/table tops or door knobs or other parts of furniture that are considered as high-touch surface in a patient’s environment”. Sheets of copper alloy, and stainless steel were cut into 5 x 5 cm coupons. All coupons were cleaned with 70% ethanol and exposed to ultraviolet light for ~30 minutes before use.

### Human Coronavirus (hCoV)

A laboratory strain of hCoV 229E was purchased from ATCC (ATCC® VR-740™) and cultured in our laboratory. Clinical specimens (Nasopharyngeal swab in Universal Transport Medium) tested positive for hCoV-229E, hCoV-OC43 and SARS-CoV-2 were also used in this study.

### Recovery of hCoV RNA from testing surfaces using RT-qPCR

#### Sample preparation

The effect of surface materials on hCoVs RNA was analyzed at 0, 1, 3 and 5 minutes of exposure time at room temperature. Briefly, 50 μl of hCoV-229E laboratory strain in MEM medium supplemented with 5% FBS (neat and 1:10 dilution) or 50 μl suspension of clinical specimen tested positive for hCoV-229 E, hCoV-OC43 and SARS-COVID-2 was placed on the different types of surfaces using a sterile pipette. Samples were collected from CSC by deep scraping the surface with a sterilized needle and a sterile swab. Virus spiked on the surfaces of stainless steel sheets, copper sheets and sterile polystyrene were recovered by a sterile pipette.

#### RNA extraction

Viral RNA was extracted from samples collected from test surfaces using the MagaZorb® total RNA Mini-Prep Kits (Promega, Madison, WI) according to the manufacturer’s instructions. One hundred μL samples were mixed with 250 μL of lysis buffer and 85 μL of 100% isopropanol. The resulting solution mixture was vortexed for 5 seconds and the lysate was transferred to a Minicolumn and centrifuged at 14,000 x *g* for 30 seconds, followed by a first wash with 500 μL of RNA wash solution, centrifuged at 14,000 x *g* for 30 seconds and then a second wash with 300 μL of RNA wash solution and centrifuged at 14,000 x *g* for 2 minutes. In the final step, 100 μL samples were used and nucleic acids were eluted with 50 μL of elution buffer. The RNA extracts were stored at -80°C until further analysis.

#### RT-qPCR assay

SARS-CoV-2 detection and quantification was performed with primers and probes targeting genes RdRP, and E as previously described by others [15,16] which are summarized in Table 1. RT-qPCR was performed using the ABI 7500 Sequence detection system with software version 1.2.3 (Applied Biosystems, USA). Amplification and detection were carried out in a final volume of 10 μL reaction mixture with 5 μL extracted RNA, 2.5 μL of 4 x TaqMan^TM^ Fast Virus 1-Step RT-PCR Master Mix (Thermo Fisher Scientific Baltics UAB, Vilnius, Lithuania), 0.4 μL of 20 μM (SARS-CoV-2); 0.4 μL of 10 μM (h229E) each forward and reverse primers, 0.2 μL of 10 μM TaqMan probe (Applied Biosystems, Foster City, CA, USA), and 1.5 μL PCR H_2_O. Amplification and detection were performed under the following conditions: an initial reverse transcription at 50°C for 5 minutes followed by PCR activation at 95°C for 20 seconds, and then 45 PCR amplification cycles (denaturation at 95°C for 3 seconds and annealing at 60°C for 30 seconds). A threshold of 0.05 was set for data analysis. A Ct value of <40 was used as a cut-off for detection of viral RNA. Samples were tested in duplicate for each gene.

**Table 1:** Primers and probes for RT-qPCR.

Target	Primer/Probe	Sequence (5’→3’)	Reference
E gene	COVID19_E_For_V2	GAG ACA GGT ACG TTA ATA GTT AAT AGC G	[16]
	COVID19_E_Rev_V2	CAA TAT TGC AGC AGT ACG CAC AC	
	COVID19_ E-Probe	*FAM*-CTA GCC ATC CTT ACT GCG-*MGB-NFQ*	
229E M gene	229E-Forward	TTC CGA CGT GCT CGA ACT TT	[17]
	229E-Reverse	CCA ACA CGG TTG TGA CAG TGA	
	229E-Probe	*FAM*-TCC TGA GGT CAA TGC A-*MGB-NFQ*	
OC43 M gene	OC43-Forward	ATGTTAGGCCGATAATTGAGGACTAT	[17]
	OC43-Reverse	AATGTAAAGATGGCCGCGTATT	
	OC43-Probe	*FAM*-CATACTCTGACGGTCACAAT- *MGB-NFQ*	
RdRp gene	RdRP_WuCoV-For_qPCR	TTTTAACATTTGTCAAGCTGTCACG	[16]
	RdRP_WuCoV-Rev_qPCR	GTTGTAAATTGCGGACATACTTATCG	
	RdRP_WuCoV_Prb_qPCR	CACTTTTATCTACTGATGGTAAC *MGB-VIC*	
Salmon DNA	sDNA-Forward	GGT TTC CGC AGC TGG G	[18]
	sDNA-Reverse	CCG AGC CGT CCT GGT C	
	sDNA-Probe	*VIC*-AGT CGC AGG CGG CCA CCG T-*TAMRA*	

An appropriate amount of salmon DNA identified at Ct = 30 using qPCR was used for monitoring inhibition of RT-qPCR as previously described [17]. Briefly, 5 μl salmon DNA (Ct = 30) was added into a sample followed by RNA extraction and qPCR for detection of salmon DNA. The qPCR for salmon DNA was carried out in a final reaction volume of 10 μL containing 5 μL of 2 x Taqman Fast Universal PCR Master Mix, 0.5 μL of primer/probe mix (18 μM of each primer and 5 μM probe listed in Table 1), 2 μL of PCR water, and 2.5 μL of RNA extract. Thermocycler conditions were initial denaturation for 20 seconds at 95°C, followed by 45 PCR amplification cycles of 3 seconds at 95°C and 30 S at 60°C. The presence of inhibitory effect was defined as a delay of at least 3 cycles as compared to a distilled water control spiked with the same concentration of salmon DNA. A negative control of distilled water was included in each RNA extraction and RT-qPCR runs.

### Inactivation of hCoV in culture

#### Cell culture

Medical Research Council cell strain 5 (MRC-5) were purchased from ATCC. Cells were cultured using Modified Eagle Medium (MEM) supplemented (Sigma-Aldrich) with 10% fetal bovine serum (FBS), 2 μM/mL of L-Glutamine and 100 μg/mL of Gentamicin (Gibco; ThermoFisher). The cells were incubated in a 37°C in a 5% CO_2_ incubator. When the cells reached 80% confluency, they were used for the inactivation test of the viruses on the different material surfaces.

#### Viral culture and determination of inactive effectiveness

The effectiveness of CSC on the disintegration of viral RNA was studied using clinical samples with SARS-CoV-2 infection and the effect of CSC on viral infectivity was demonstrated with hCov-229E in MRC-5 cell culture. Since working with SARS-CoV-2 viral culture requires BSL-3 laboratory conditions, it has been highly suggested that the use of surrogates of human SARS-CoV-2 to study and understand outcomes and mechanisms on survival and inactivation under various conditions [19]. For this reason, we chose to culture hCoV-229E as a surrogate of human SARS-CoV-2 in the same family of human coronaviruses. The surrogate has similar properties to that of virulent hCoVs responsible for MERS and SARS [20].

Test surfaces were contaminated with 50 μl of hCoV 229E (∼8·9 log unit of 50% tissue culture infectious dose assay [TCID_50_] per mL) suspension using a sterile pipette. Samples were collected by deep scraping and a sterile swab. Virus spiked on the surfaces of stainless steel sheets, copper sheets and sterile polystyrene were recovered by a sterile pipette at 0, 0.5, 1, 3, 5 and 60 minutes of exposure time. Viral titers were determined using the TCID50 method in MRC-5 cells. Cells were inoculated with 10-fold serial dilutions of samples collected from the testing surfaces in 100 μL MEM (Sigma-Aldrich) supplemented with 10% FBS, 2 μM/mL of L-glutamine and 100 μg/mL of gentamicin in 96 well pates and incubated for 1 h at 37°C and 5% CO_2_ atmosphere with 95% humidity. For each dilution step, four wells of replicates were inoculated. After one hour of incubation the inoculum was removed and 300 μL of maintenance MEM supplemented with 1% FBS, 2 μM/mL of L-glutamine and 100 μg/mL of gentamicin was added to each well. Cells were incubated for seven days and scored for cytopathic effect (CPE) on a daily basis. hCoV 229E induced CPE of infected cells was determined by observing rounded, detached cells in close association to each other. Evidence of inactivation was determined by absence of CPE in MRC-5 cells, indicating loss of infectivity. The TCID50 was calculated via the Reed-Muench formula [18]. The log-value was a measure of the effectiveness of the disinfectant in inactivating the virus. This value was calculated by the following formula: Inactivation log-value = log N_0_−log N_x_ where N_0_ is the TCID_50_ of the sterile polystyrene group, and N_x_ is the TCID_50_ of the CSC group.

### Statistical analysis

All the graphs and statistical analyses were performed using GraphPad Prism software version 8.0 for windows (GraphPad Software, San Diego, CA, USA). To test whether the numerical data were normally distributed, Shapiro-Wilk normality tests were applied. The comparison of means between different groups of numerical variables was performed using non-parametric two-way ANOVA followed by the Friedman test, and a p value less than 0.05 was considered as statistically significant.

## Results

### Recovery of SARS-CoV-2 from test surfaces

As described in the materials and methods section, all test surfaces including sterile polystyrene, stainless steel and copper coupons were cleaned with 70% ethanol and exposed to ultraviolet light for ~30 minutes before use. Prior to inoculating the test surfaces with viral preparation, the surfaces, including CSC, stainless steel, copper and sterile polystyrene were also tested for the presence of hCoV-229E RNA using RT-PCR. All surfaces tested negative for hCoV-229E. Next, we tested the recovery of a laboratory strain hCoV-229E at time points 0 (immediately after exposure ~10 seconds), 1, 3 and 5 minutes after placing the viral suspension on these surfaces. To enhance recovery of the virus from the CSC surface, virus was recovered from CSC using a scratch and swab collect method, and a pipette was used to collect samples from stainless steel, copper sheets and sterile polystyrene. The recovery rate (%) was calculated by dividing the amount of virus detected after placement on various surfaces by the amount of virus detected in the original used viral preparation sample x 100. The recovery was 94.4% (neat) and 93.6% (1:10 dilution) for stainless steel; 92.6% (neat) and 91.2% (1:10 dilution) for copper sheets, and 96.8% (neat) and 97.3% (1:10 dilution) for the sterile polystyrene. The recovery of viral RNA for CSC immediately after exposure (~10 seconds) was reduced with notable increase of cycle threshold (Ct) values. To determine whether the low recovery of viral RNA from the CSC surface was caused by inhibitory effect of increase sodium chloride concentration on DNA amplification, an internal quality control of salmon DNA was added to the samples before and after the viral RNA extraction. No increases in the Ct values were observed for salmon DNA indicating that CSC has no inhibitory effect on the PCR reaction.

### Time dependent effect of CSC on viral RNA using RT-qPCR

The time-dependent effect of CSC on the RNA of hCoV-229E laboratory strain was tested at exposure times 0 (~10 seconds), 1, 3 and 5 minutes. The Ct values in the CSC group were higher than steel, copper and sterile polystyrene at all time points. Compared to the sterile polystyrene, approximately 2 to 3- log10 reductions in detectable viral RNA was achieved by CSC at 0 minute (~10 seconds) post-exposure, suggesting that CSC is effective at reduction of laboratory strain hCoV-229E RNA within a short time of exposure (**Table 2**). Similarly significant log_10_ reduction in viral RNA genomic level was observed for CSC at 0 minute when compared to steel, copper and sterile polystyrene when tested with clinical samples that had tested positive for hCoV-229E and hCoV-OC43; *p*<0.001 (**Table 3**). In addition, a significant (*p*<0.001) 3 to 4-log_10_ reduction was observed at 0 minute when CSC was exposed to clinical samples tested positive for SARS-CoV-2 (RdRp and E gene) (**Table 3**). However, no obvious time-dependent reduction of viral RNA was observed after exposure to CSC suggesting that the maximal effectiveness of CSC may have already been achieved immediately after exposure.

**Table 2:** Effect of CSC against laboratory hCoV-229E strain at different time points.

Surface Tested	Ct values			
	Time points (minutes)			
	0	1	3	5
Sterile polystyrene (Control)	18.54±0.62	16.40±0.07	16.59±0.05	16.41±0.07
CSC	28.75±0.03*	30.43±0.25*	31.55±0.59*	32.07±0.12*
Copper	18.43±0.16	16.38±0.05	16.43±0.17	16.94±0.43
Stainless steel	20.42±0.02	16.0±0.07	16.67±0.12	17.11±0.84

Results are represented as means of replicates ±SD between the replicates.

*p <0.05 compared to Sterile polystyrene (Control) at each time point.

**Table 3:** The effectiveness of CSC against SARS-CoV-2 in clinical samples by RT-qPCR.

Surface tested	Ct value			
	Time points (minutes)			
	0	1	3	5
**Sterile polystyrene (Control)**				
***hCoV -229E***				
Sample 1	24.40±0.13	23.85±0.01	24.31±0.34	24.75±0.54
Sample 2	23.44±0.13	22.74±0.04	23.75±0.00	23.20±0.25
***hCoV-OC43***				
Sample 1	22.32±0.19	22.01±0.07	22.11±0.03	23.76±0.31
Sample 2	23.07±0.21	22.76±0.05	23.23±0.04	23.81±0.28
***RdRp gene***				
Sample 1	19.49±0.07	19.33±0.05	19.29±0.07	19.10±0.04
Sample 2	19.50±0.06	18.75±0.01	19.85±0.07	19.53±0.20
***E gene***				
Sample 1	20.08±0.02	19.95±0.01	19.93±0.05	19.76±0.08
Sample 2	17.34±0.14	16.91±0.04	18.26±0.01	17.58±0.08
**CSC**				
***hCoV -229E***				
Sample 1	33.25±0.18*	35.83±1.37*	35.68±0.91*	36.45±0.21*
Sample 2	34.29±0.01*	33.79±0.17*	35.73±0.81*	41.42±1.54*
***hCoV-OC43***				
Sample 1	31.75±0.45*	32.60±0.12*	32.79±0.00*	31.44±0.09*
Sample 2	33.84±0.30*	32.77±0.03*	33.32±0.24*	35.13±0.03*
***RdRp gene***				
Sample 1	29.89±0.09*	31.63±0.01*	30.53±0.08*	31.73±0.18*
Sample 2	29.83±0.13*	30.54±0.45*	30.92±0.82*	31.11±0.13*
***E gene***				
Sample 1	30.37±0.24*	30.32±0.16*	31.04±0.14*	32.64±0.12*
Sample 2	28.31±0.10*	30.38±0.23*	29.60±0.36*	30.52±0.10*
**Copper**				
***hCoV -229E***				
Sample 1	24.74±0.20	24.57±0.08	25.75±0.10	24.84±0.12
Sample 2	23.60±0.89	22.33±0.41	22.16±0.34	22.00±0.31
***hCoV-OC43***				
Sample 1	22.21±0.16	21.72±0.15	21.93±0.02	24.21±0.24
Sample 2	23.43±0.02	23.31±0.07	23.34±0.13	23.46±0.34
***RdRp gene***				
Sample 1	19.29±0.12	19.31±0.05	20.45±0.03	19.18±0.04
Sample 2	20.34±0.08	19.54±0.06	19.78±0.06	19.58±0.13
***E gene***				
Sample 1	19.77±0.13	16.87±0.07	19.63±0.01	19.76±0.01
Sample 2	18.20±0.04	17.65±0.06	17.90±0.10	17.72±0.18
**Stainless steel**				
***hCoV -229E***				
Sample 1	25.09±0.18	23.88±0.01	24.01±0.91	24.14±0.13
Sample 2	22.22±0.32	21.73±0.23	21.92±0.04	23.69±1.65
***hCoV-OC43***				
Sample 1	21.13±0.19	24.34±0.91	21.66±0.08	23.85±0.24
Sample 2	23.15±0.21	23.59±0.04	23.18±0.14	23.46±0.34
***RdRp gene***				
Sample 1	19.23±0.07	19.15±0.12	19.17±0.09	19.17±0.03
Sample 2	19.17±0.23	19.13±0.19	19.24±0.04	19.38±0.14
***E gene***				
Sample 1	19.77±0.13	16.85±0.07	19.63±0.00	19.76±0.01
Sample 2	17.27±0.04	17.47±0.05	17.90±0.10	17.72±0.18

Results are represented as means of replicates ±SD between the replicates.

*p <0.05 compared to Sterile polystyrene (Control) at each time point.

### Inactivation of hCoV-229E by CSC using cell culture and TCID50

**Fig 1:**
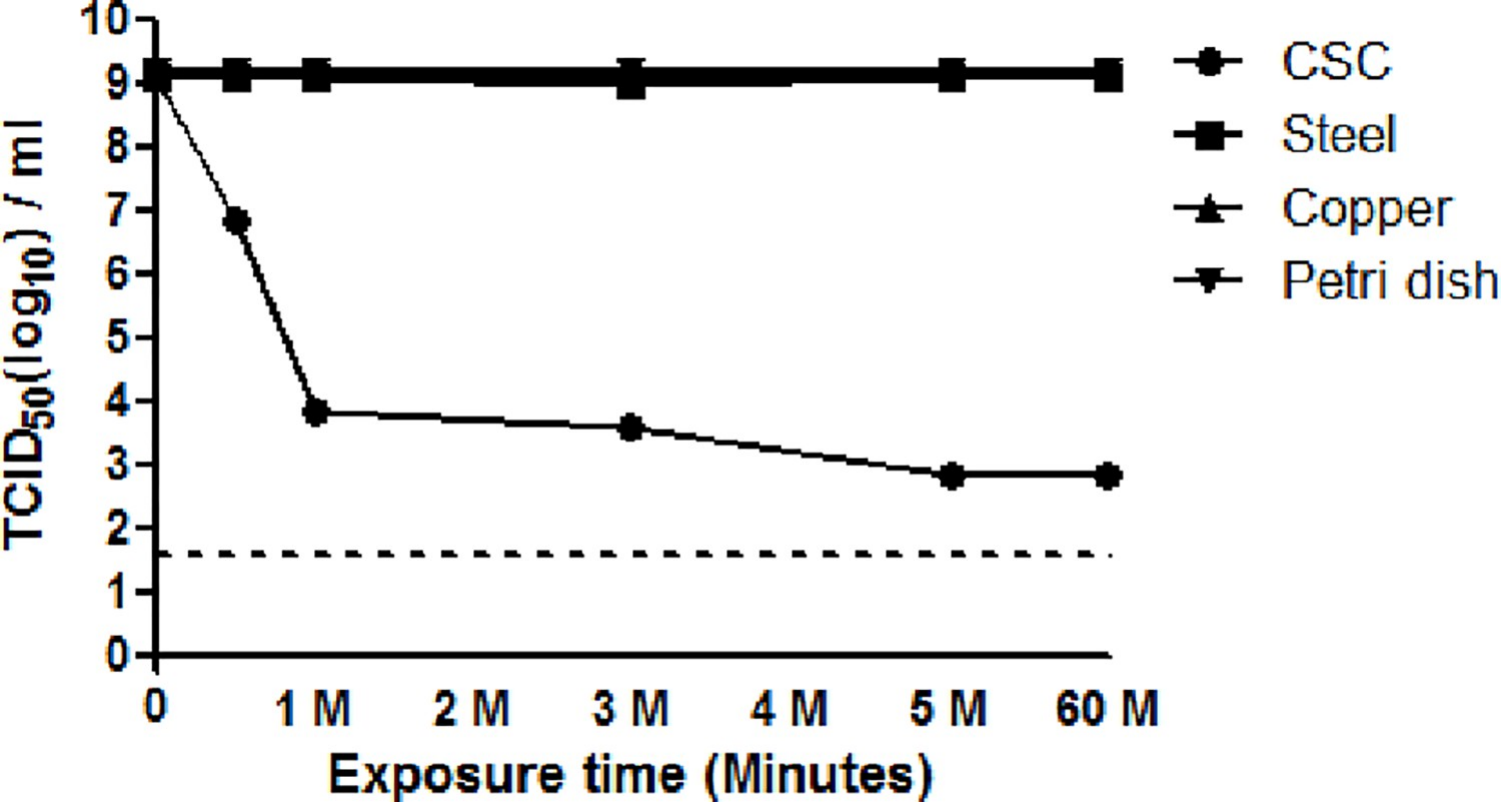
Effect of CSC on the infectivity of hCoV 229E was exposed to CSC surface and the samples were collected at each time point and titrated in MRC-5 cells. The results shown are representative of duplicates. Control groups (steel, copper and sterile polystyrene) were treated identically at each time point. Samples were titrated in MRC-5 cells using four replicates. The dotted line denotes the limit of detection of the assay.

**Fig 2:**
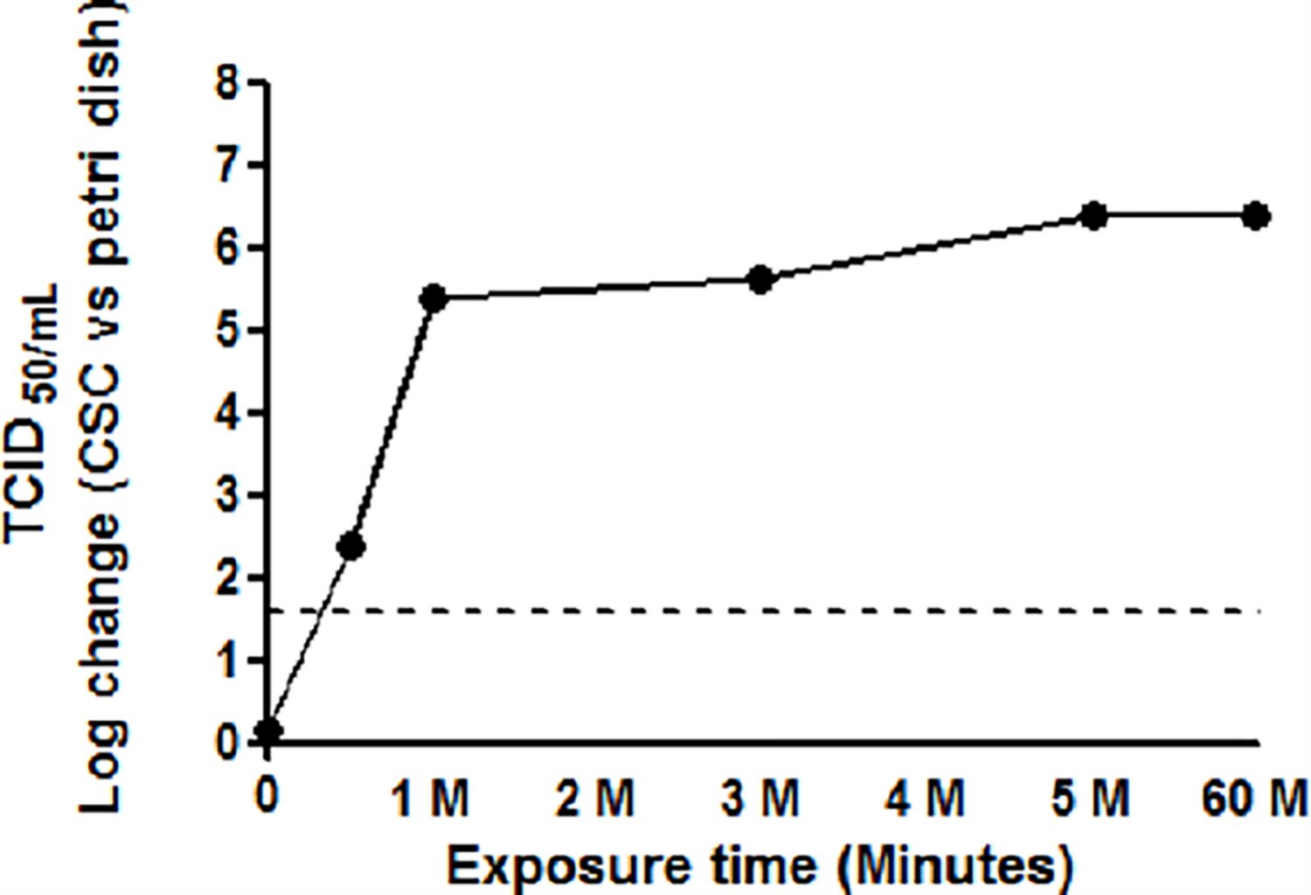
Effect of CSC in inactivating hCoV 229E compared to the sterile polystyrene group. The inactivation log change value was calculated by subtracting the TCID_50_ value of sterile polystyrene from TCID_50_ value of CSC group collected at each time point. The dotted line denotes the limit of detection.

To examine the viral inactivation effect of CSC, hCoV-229E virus stocks were exposed to CSC for varying amounts of time and the recovered suspension were placed in 96-well tissue culture plates, as indicated in **Fig 1**. Exposure of hCoV-229E to CSC resulted in 2.38 log reduction at 0.5 minute compared to time 0 control with time-dependent inactivation up to 5 minonnutes (**Fig 1**). The virus was almost completely inactivated and close to the detection limit (≤1.58 TCID_50_ (log_10_) per ml) within 5 minutes. In contrast, steel and copper exposure demonstrated no significant effects on viral infectivity for hCoV-229E after an hour of exposure (**Fig 1**). Compared to sterile polystyrene, CSC demonstrated a 6 log_10_ decrease in cultivable virus (**Fig 2).** To determine the effect of CSC on the cultured cells alone during the viral infection experiments, an internal quality control of CSC alone were exposed to cultured cells in triplicates. No side-effect of CSC was observed on the cultured cells, indicating that CSC has no inhibitory or cytotoxic effect on the cultured cells during the viral infection.

## Discussion

Human coronaviruses can remain infectious on inanimate surfaces for several days [21], surface disinfection with chemicals is an important step in preventing contact transmission of these viruses. Generally, coronaviruses are not highly resistant to disinfectants and can also be inactivated by heat and ultraviolet irradiation [22]. Alcohol based hand rubs have been labelled to have immediate strong antiviral activity [21] against different enveloped viruses such as orthopoxvirus, influenza A virus [23,24], herpes simplex virus type 1 and 2 [24], Newcastle disease virus, togavirus [25], hepatitis B virus [26–28], human immunodeficiency virus [24,29,30], and SARS-CoV [31]. Disinfectants such as ethanol (70–95%) or isopropanol (50–100%) inactivates SARS-CoV-2 [32]; however, it must be used with adequate ventilation and require a drying time of at least 30 min and can cause acrylic resin degradation when disinfecting synthetic resin materials. High level of environmental contamination of SARS-CoV-2 RNA and infectious virus has been reported in severely immunocompromised patients with severe COVID-19 [33]. Thus, disinfection of surfaces is important in the prevention of the spread of SARS-CoV-2 on inanimate surfaces. Previously a new, inexpensive and safe material made of CSC was shown to inactivate bacteria and fungi upon contact [12,13]. In this study, we examined the effect of CSC on SARS-CoV-2 and surrogate virus hCoV-229E and hCoV-OC43. The CSC surface achieved a significant reduction in viral RNA genome of all three viruses. There was an approximately 3 to 4 log_10_ reductions in detectable SARS-CoV-2 RNA after exposure to CSC as compared to sterile polystyrene, and 2 to 3 log_10_ reductions in detectable viral RNA compared to copper and steel within 0.5 minute.

In our study CSC only showed 3 to 4 log 10 reduction of SARS-CoV-2 rather than nearly all of them because we have used an inoculum with high viral titre in our study to represent a worst-case scenario of SARS-CoV-2 persistence on a contaminated surface. Clinical data from mild and severe cases has revealed a range of data, with median initial viral loads of 5.11 and 6.17 log_10_ copies of viral RNA per ml in mild and severe cases, respectively [34].” If we have used a low titre to mimic more closely a casual contact scenario, a decrease in sensitivity would be expected and thus duration of viable virus detection may have been reduced. In most of disinfection studies, 4 log 10 reduction of infectivity of any agents has been considered a significant decrease of infection.

The present study results are consistent with previous investigations on surface disinfectants which demonstrated that sodium hypochlorite solution (effective concentration, 0.21%) achieved rapid activity against MERS-CoV or hCoV -229E and hCoV-OC43 viral nucleic acid [10,35]. However, because of the high alkaline nature of sodium hypochlorite, it has a strong corroding effect [36] on acrylic resins and it is unknown if sodium hypochlorite acts preferably against the viral genome or viral inactivation. Another study confirmed that benzalkonium chloride, a representative quaternary ammonium salt (QAC) disinfectant, can inactivate SARS-CoV-2. However, the effect of benzalkonium chloride on SARS-CoV-2 has been controversial [37]. The US CDC has not yet approved benzalkonium chloride because the supporting research reported that 0.05–0.2% benzalkonium chloride was less effective against MERS-CoV and hCoV-229E [38]. It has been reported that benzalkonium chloride-based product (Dettol Hospital Concentrate) was active against human coxsackievirus but not effective against HCoV and other non-enveloped viruses [39]. Another QAC-based disinfectant, Di-N-decyl dimethyl ammonium chloride (DNC), inactivated SARS-CoV-2 at 5000 mg/L for 30 min with reduction of 3.25 log10 regardless of the type of biological load [10]. A possible explanation for inconsistent inactivation of viruses was that the antimicrobial activity of QAC compounds was selective due to the presence of molecular alkyl chains and its interaction with targeting microbes [40]. However, there were some reports of QAC compounds with toxic to aquatic and terrestrial organisms [15,35,41]. Although many formulations of QAC are still widely used in liquid-based disinfectants and have shown to be effective against non-enveloped and enveloped viruses, there was limited laboratory data available on the inactivation efficacy of QAC against SARS-CoV-2. Since our study was aimed to evaluate the effect of surface-derived disinfection of CSC hard surface on SARS-CoV-2 and other viruses, we did not use QAC liquid disinfectant as an active control.

The results from our study support that CSC could effectively target the viral genome of SARS-CoV-2 resulting in decreased detection in a very short time. In addition, the inactivation of hCoV-229E, a surrogate virus with high genetic homology, by CSC supports its use as a surface disinfectant for SARS-COV-2. A perfect antimicrobial surface would be fast acting and inexpensive, as well as non-toxic to environments. The immediate antiviral effect of CSC, the extremely low price and the high availability of sodium chloride (the sole ingredient in CSC surfaces) and its anti-bacterial and anti-fungal activity support the use of CSC as a surface disinfectant.

To the best of our knowledge, this study is the first to use the hCoV strains and SARS-CoV-2 as a comprehensive comparative evaluation of the effect of CSC on these viruses. Limitations of this study are that the mechanism of degradation of SARS-CoV-2 RNA after contact was not directly studied and only surrogate hCoV-229E was assessed in terms of decrease in infectious virus. The physical properties and mechanism of CSC surfaces need to be further studied and characterized to optimize its application.

In summary, we identified that CSC is effective in degrading SARS-CoV-2 RNA derived from clinical samples in the same way for hCoV RNA from cultured hCoV 229E, which was supported by inactivation of hCoV 229E virus in cell culture.
